# Increased Level of IFN-γ and IL-4 Spot-Forming Cells on ELISPOT Assay as Biomarkers for Acute Graft-*Versus*-Host Disease and Concurrent Infections

**DOI:** 10.3390/cells1020061

**Published:** 2012-04-30

**Authors:** Masahiro Hirayama, Eiichi Azuma, Yoshihiro Komada

**Affiliations:** Department of Pediatrics and Cell Transplantation, Mie University Graduate School of Medicine, 2-174 Edobashi, Tsu, Mie 514-8507, Japan; Email: hirayama@clin.medic.mie-u.ac.jp (M.H.); komada@clin.medic.mie-u.ac.jp (Y.K.)

**Keywords:** ELISPOT, acute GVHD, HSCT, IFN-γ, biomarker

## Abstract

Acute graft-*versus*-host disease (aGVHD) remains a significant cause of morbidity and mortality after allogeneic hematopoietic stem cell transplantation. Infections may coexist and in certain circumstances aggravate aGVHD. It was described that type 1 as well as type 2 cytokines are important mediators of aGVHD. We measured spot-forming cells (SFCs) for interferon (IFN)-γ, interleukin (IL)-4, IL-10, and IL-17 in unstimulated peripheral blood from 80 patients with hematological disorders who underwent allogeneic hematopoietic stem cell transplantation by using the enzyme-linked immunospot (ELISPOT) assay that reflects the ongoing *in vivo* immune status. A serial monitoring showed that both type 1 and type 2 cytokine SFCs were correlated with aGVHD activity. The numbers of IFN-γ and IL-4 SFCs in patients with grade II-IV aGVHD were significantly higher than those in patients with grade 0 and/or I aGVHD. Elevation of IFN-γ and IL-4 SFCs was significantly correlated with the severity of aGVHD, but not with infection itself, e.g., cytomegalovirus infection. Cytokine SFCs are clinically relevant biomarkers for the diagnostic and therapeutic evaluation of aGVHD and concurrent infection.

## 1. Introduction

Acute graft-*versus*-host disease (aGVHD) remains a potentially fatal complication of allogeneic hematopoietic stem cell transplantation (HSCT). Susceptibility to infection has posed one of the most formidable obstacles in the clinical management of patients undergoing allogeneic HSCT especially from the preengraftment to the immediate postengraftment periods [1]. In addition, infections may aggravate aGVHD after allogeneic HSCT [2]. aGVHD is initiated by the action of donor-derived T cells which have been suggested to polarize into type 1 T cells after being stimulated with interleukin (IL)-12 from antigen-presenting cells [3]. The balance between type 1 cytokines and type 2 cytokines is hypothesized to govern the extent to which all cell-mediated immune responses and inflammatory responses develop after allogeneic HSCT.

So far, great attention has been paid to the detection of aGVHD before and after HSCT. Some serum markers, such as the levels of tumor necrosis factor-α, soluble Fas, IL-2Rα, IL-8, IL-10, hepatocyte growth factor, and CCL8 have been reported to be useful indicators of aGVHD [4,5,6,7,8]. Polymorphism of the IL-10 gene [9] and transforming growth factor ß1 gene [10] are associated with aGVHD. Cytotoxic T lymphocyte precursor (CTLp) and helper T lymphocyte precursor (HTLp) frequency analysis has been shown to predict aGVHD [11]. On the other hand, failure to detect these cytokines in the serum has also been reported [4]. aGVHD was not differentiated from infection or fatal complications by the assessment of cytokines [12]. CTLp and HTLp frequency analysis has been reported to be not necessarily for predicting aGVHD [13]. Thus, there are contradictory results among these reports and there still remain problems with attempts to use these parameters as reliable and sensitive biomarkers of aGVHD. 

Enzyme-linked immunospot (ELISPOT) assay may circumvent the limitations described above. ELISPOT assay permits the *ex vivo* identification of cells actively secreting cytokines. In fact, Tanguay *et al*. reported high specificity combined with 10–200 times higher sensitivity than that of conventional ELISA [14]. However, there are some disadvantages of the ELISPOT assay. It is not able to detect simultaneous multiparametric measurement unlike intracellular cytokine staining (ICS) unless cell-specific separations are performed before the assay. It takes 1–2 days for incubation and counting. On the other hand, ICS also has disadvantages that include lower sensitivity than ELISPOT assay and the requirement for *in vitro* stimulation, indicating that ICS detects synthesized but not secreted cytokines [15]. Nevertheless, an important advantage of ELISPOT assay is that it is a direct measurement of a type 1 and type 2 cell-mediated immune response.

Given the central role of alloreactive T cells as aGVHD effector cells, we hypothesized that circulating cytokine spot-forming cells (SFCs) could be biomarkers of aGVHD. Therefore, we measured cytokine SFCs after the emergence of aGVHD, with the rationale that cytokine SFCs by ELISPOT assay may reflect the ongoing *in vivo* immunological status after transplantation. In this study, we used the ELISPOT assay to detect and enumerate cells producing IFN-γ and IL-4 to evaluate the influence of type 1 and type 2 T-cell cytokines during infections and/or aGVHD.

## 2. Materials and Methods

### 2.1. Patient Characteristics

Eighty patients who underwent allogeneic HSCT between 1996 and 2010 were included in this study. Twenty-six patients received bone marrow from unrelated donors, 34 patients received bone marrow from related donors, 9 patients received peripheral blood stem cell from related donors, and 11 patients received cord blood from unrelated donors. Patients’ demographics are shown in Table 1. Underlying diseases were acute lymphoblastic leukemia (n = 21), acute myeloid leukemia (n = 21), chronic myeloid leukemia (n = 11), aplastic anemia (n = 12), myelodysplastic syndrome (n = 4), malignant lymphoma (n = 5), advanced neuroblastoma (n = 4), and others (n = 2; Kostmann syndrome and Wiskott-Aldrich syndrome). In human leukocyte antigen (HLA) mismatched transplants (4/6, 3/6), stem cell source was cord blood, resulting in grade 0-I aGVHD. The mean age of the patients was 14.8 years (range, 1–61). 

**Table 1:** Patient characteristics.

Characteristic		aGVHD grade 0 (n = 49)	aGVHD grade I-IV (n = 31)	P
Median age (range)		15 (1–61)	14 (1–49)	NS*
Male: Female		33:16	19:12	NS#
Donor type				
	Related	23	13	NS#
	Unrelated	26	18	
Stem cell source				
	Bone marrow	37	22	
	Peripheral blood	5	5	NS†
	Cord blood	7	4	
GVHD prophylaxis				
	Cyclosporin A ± MTX	40	24	NS#
	Tacrolimus ± MTX	9	7	
Conditioning regimen				
	TBI based	26	19	
	BU based	15	9	NS†
	Others	8	3	
HLA histocompatibility				
	6/6	38	26	
	5/6	6	4	NS†
	4/6, 3/6	5	1	
Pretransplant CMV serostatus				
(Donor/recipient)				
	+/+	24	14	NS†
	−/+	9	8	
	+/−	11	4	
	−/−	5	5	
Posttransplant CMV antigenemia				
	Positive	16	10	NS#
	Negative	33	21	

Abbreviations used: MTX, methotrexate; TBI, total body irradiation; BU, busulfan; CMV, cytomegalovirus; NS, not significant (significant level = 0.05, NS*: determined using 2-tailed Mann-Whitney test, NS^#^: 2-tailed Fisher exact test, NS^†^: Kruskal-Wallis test).

### 2.2. Evaluation of Events and Sample Collection

aGVHD was proved histopathologically by biopsy. The grading of aGVHD was determined according to clinical criteria [16]. Cytomegalovirus (CMV) infection was diagnosed on the basis of clinical symptom, histopathology, and antigenemia [17,18]. 

Peripheral blood samples were collected from patients at 3 (early engraftment period), 6, and 10 (late engraftment period) weeks after transplantation. Informed consent was obtained from all participants and approval for the study was obtained from the institutional ethics committee review board. Peripheral blood mononuclear cells (PBMCs) were separated from heparinized peripheral blood using Ficoll-Hypaque density gradient centrifugation.

### 2.3. ELISA

Plasma IFN-γ, IL-4, and IL-10 (BD Pharmingen, San Diego, CA) were measured using ELISA kits according to the manufacturer’s instructions as described previously [19]. The detectable levels of IFN-γ, IL-4, and IL-10 were all > 4 pg/mL.

### 2.4. ELISPOT Assay

ELISPOT assay was undertaken as described previously [20]. Briefly, ELISPOT plates (Millipore Corp., Bedford, MA, USA) were coated with anti-human IFN-γ, IL-4 (Mabtech AB, Stockholm, Sweden), IL-10, or IL-17 (BD Pharmingen, San Diego, CA, USA) monoclonal antibody overnight at 4 °C. The plates were washed three times and incubated for 2 h with RPMI-1640 containing 10% fetal bovine serum. Freshly isolated, unstimulated PBMCs were added at the concentration of 50,000 cells per well. As a positive control, PBMCs were stimulated with Phorbol 12-myristate 13-acetate (PMA) (100 ng/mL) and Ionomycin (1 µg/mL) (Sigma-Aldrich, Tokyo, Japan). The plates were incubated for approximately 40 h at 37 °C and 5% CO_2_ in a humid atmosphere. The cells were removed and the plates were developed with a second biotinylated monoclonal antibody to human IFN-γ, IL-4 (Mabtech AB), IL-10, or IL-17 (BD Pharmingen), then washed six times. The plates were developed with streptavidin-alkaline phosphatase and colorimetric substrate (Mabtech AB). The number of resulting spots was counted with an ImmunoSpot Analyzer using acquisition and analysis software (Carl Zeiss, Tokyo, Japan). Data were obtained from triplicate samples and standard error was less than 10%.

### 2.5. Statistical Analysis

Data are presented as means ± standard deviations (SD). Student’s t-test was used for analysis. Fisher’s exact test, Mann-Whitney test, and Kruskal-Wallis test were used for contingency table analysis. P-value less than 0.05 was considered statistically significant. All statistical analyses were performed using Statview Version 5.0 (SAS Institute Inc., Cary, NC, USA).

## 3. Results

### 3.1. Spot-Forming Cells in Patients Who Underwent Allogeneic HSCT

**Figure 1:**
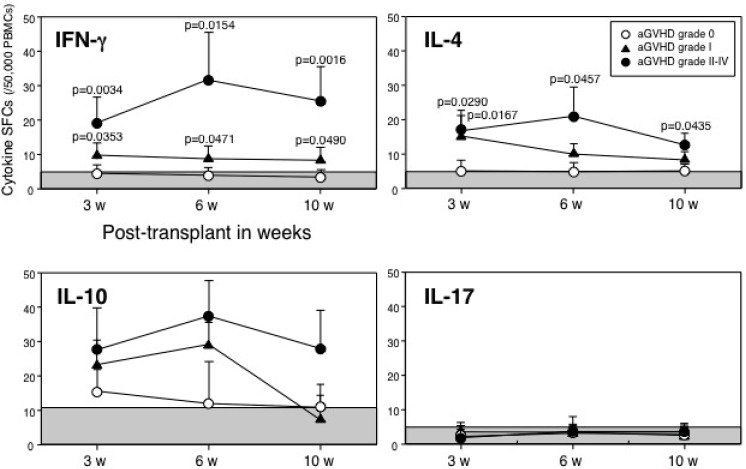
Kinetics of cytokine spot-forming cells (SFCs) in patients with acute graft-*versus*-host disease (aGVHD). Analysis of time course of the numbers of cytokine SFCs. Numbers of samples in IFN-γ at 3, 6, and 10 weeks after transplantation were 41, 41 and 35 on grade 0 aGVHD, 11, 12 and 9 on grade I, and 17, 15 and 15 on grade II-IV, respectively. Similarly, numbers of samples in IL-4 were 39, 40 and 33 on grade 0; 10, 11 and 8 on grade I; 16, 14 and 14 on grade II-IV. Numbers of samples in IL-10 were 25, 23 and 21 on grade 0; 5, 6 and 4 on grade I; 10, 9 and 10 on grade II-IV. Numbers of samples in IL-17 were 12, 10 and 9 on grade 0; 5, 5 and 3 on grade I; 4, 3 and 3 on grade II-IV. Data are expressed as means ± SD. Each shaded area was determined from healthy donor PBMCs as the cut-off value (n = 35; mean + 2SD).

To evaluate the influence of SFCs on aGVHD, we measured the numbers of SFCs for IFN-γ, IL-4, IL-10 and IL-17 in unstimulated PBMCs of 49 patients with grade 0 aGVHD, 14 patients with grade I, and 17 patients with grade II-IV at 3, 6, and 10 weeks after transplantation. In the total 196 samples, as shown in Figure 1, IFN-γ and IL-4 SFCs at 3, 6, and 10 weeks after transplantation in patients with grade II-IV aGVHD were significantly higher than those in patients with grade 0 and/or I aGVHD. There were no significant differences in IL-10 and IL-17 SFCs among grade 0, I, and II-IV aGVHD, although IL-10 SFCs in grade II-IV aGVHD were elevated at every point after transplantation. These results indicate that not only type 1 (IFN-γ), but also type 2 (IL-4) SFCs were involved in aGVHD following HSCT. Therefore, enumeration of type 1 and/or type 2 cytokine SFCs in PBMC samples with patients after transplantation is a good indicator for evaluating aGVHD.

The number of IFN-γ, IL-4, IL-10, and IL-17 SFCs used for the cut-off value, determined from 35 healthy donors, was 5.2, 4.5, 10.8, and 4.8 spots /50,000 PBMCs (mean + 2SD), respectively.

Based on the cut-off value for IFN-γ SFCs, the true-positive rate was 97% (44/45 samples) during aGVHD in patients with grade I-IV aGVHD and the false-positive rate was 16% (25/151 samples) in patients with grade 0 aGVHD. With regard to IL-4 SFCs, the true-positive rate was 91% (41/45 samples) and false-positive rate was 23% (33/140 samples) in patients with grade 0 aGVHD.

**Figure 2:**
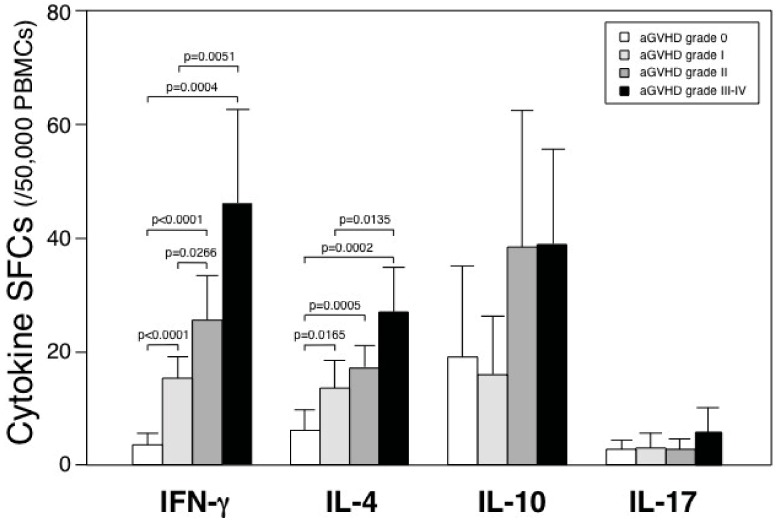
IFN-γ and IL-4 SFCs indicate severity of aGVHD. The numbers of cytokine SFCs were analyzed according to the severity of aGVHD. Numbers of samples in IFN-γ were 151, 17, 13 and 15 on grade 0, I, II and III-IV, respectively. Similarly, numbers of samples in IL-4 were 141, 17, 13, and 15; in IL-10, 87, 10, 9 and 7; in IL-17, 38, 9, 4 and 3.

Next, we analyzed the number of cytokine SFCs according to the severity of aGVHD. The number of SFCs for IFN-γ and IL-4, but not IL-10 and IL-17, significantly increased along with the higher grade of aGVHD (Figure 2). Thus, these results suggest that both type 1 and type 2 cytokine contribute to aGVHD.

### 3.2. Plasma Cytokine Levels in Patients Who Underwent Allogeneic HSCT

The plasma levels of IFN-γ, IL-4, and IL-10 were measured in 26 patients with grade 0 and 18 patients with grade I-IV aGVHD at 3, 6 and 10 weeks after transplantation (data not shown). No statistical differences in these cytokine levels were observed between the patients with grade 0 and with grade I-IV aGVHD. IFN-γ was detected in 3 out of 18 patients (16%) with grade I-IV aGVHD. Similarly, IL-4 and IL-10 were detected in 5 of 17 (29%) and 8 of 13 (61%) patients with grade I-IV aGVHD, respectively. Thus, the detection rates were relatively low for all cytokines. These results indicate that measurement of plasma cytokine may not be a useful indicator of aGVHD.

### 3.3. ELISPOT Assay Discriminates aGVHD from Infection

**Figure 3:**
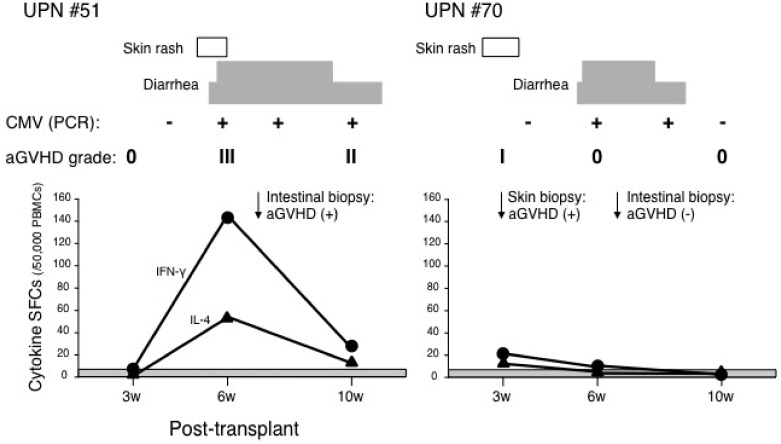
IFN-γ and IL-4 SFCs were elevated in patients with aGVHD, irrespective of CMV infection. Two representative cases developed stage 2 diarrhea with positive antigenemia for CMV. One case (UPN#51) developed intestinal aGVHD, the other case (UPN#70) did not. Shaded area is shown as cut-off value for IFN-γ SFCs.

CMV-specific immunodeficiency may persist for 3 months after HSCT [21]. CMV viremia, quantitative pp65 antigenemia, and DNA load are risk factors for the development of CMV disease, independent of aGVHD [22]. It is often difficult to differentiate CMV infection from aGVHD when they coexist. We have shown two cases developed in comparative course (Figure 3). A 16-year-old male patient (UPN#51) with acute lymphoblastic leukemia developed stage 3 skin rash on day 34 after HSCT, which was accompanied by stage 2 diarrhea. He had mild elevation of transaminases and positive CMV antigenemia. The level of IFN-γ and IL-4 SFCs increased along with the increased level of diarrhea. On day 50, intestinal biopsy proved the presence of aGVHD. In contrast, a 10-year-old male patient (UPN#70) with chronic myelogenous leukemia developed stage 3 diarrhea on day 38 after HSCT. He had mild elevation of transaminases and positive CMV antigenemia. The level of IFN-γ and IL-4 SFCs did not increased during diarrhea. On day 55, intestinal biopsy proved enteritis with CMV inclusion bodies, but not any apoptotic bodies of crypt. Thus, those representative cases demonstrated that the ELISPOT assay might discriminate aGVHD from CMV infection.

**Figure 4:**
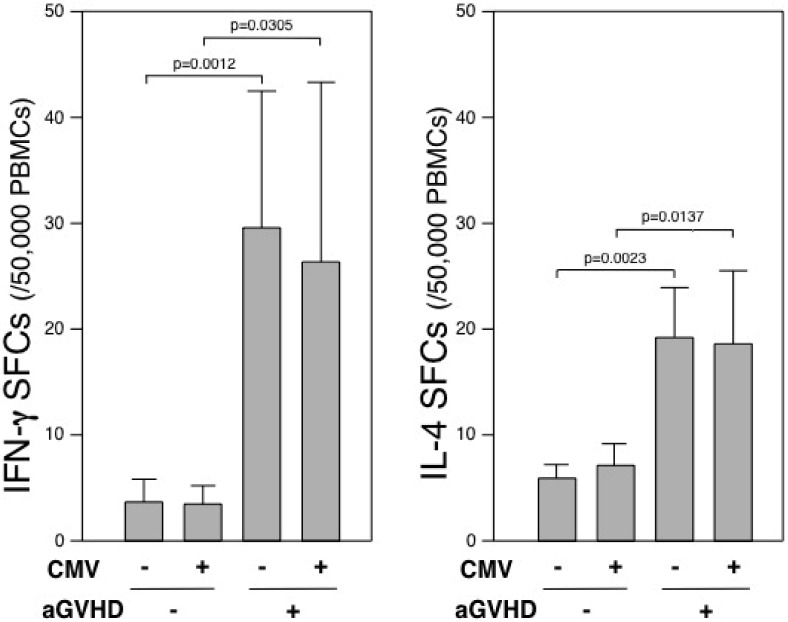
Discrimination of aGVHD from CMV infection by IFN-γ and IL-4 SFCs. The numbers of IFN-γ and IL-4 SFCs in patients with grade 0 and I-IV aGVHD were analyzed by comparing with negative or positive CMV infection. Data are expressed as means ± SD.

Recipient seropositivity for CMV, which has been reported as a prognostic factor [23], was 67% (33/49) and 70% (22/31) in grade 0 aGVHD and grade I-IV aGVHD, respectively (Table 1). In the group of grade I-IV aGVHD, the mean numbers of IFN-γ SFCs with negative (sample number = 31) or positive (n = 14) CMV antigenemia were 29.5 ± 20.8 and 26.3 ± 35.1 SFCs/50,000 PBMCs, respectively. These were significantly higher than the group of grade 0 aGVHD, irrespective of negative (n = 128; 3.7 ± 2.6 SFCs/50,000 PBMCs) or positive antigenemia (n = 23; 3.5 ± 2.6). Similarly, in the group of grade I-IV aGVHD, the mean numbers of IL-4 SFCs with negative (n = 31) or positive (n = 14) CMV antigenemia were 19.1 ± 12.5 and 18.5 ± 16.3 SFCs/50,000 PBMCs, respectively. These were significantly higher than the group of grade 0 aGVHD, irrespective of negative (n = 115; 5.9 ± 7.6 SFCs/50,000 PBMCs) or positive antigenemia (n = 23; 7.1 ± 10.5). These results indicate that enumeration of IFN-γ and IL-4 SFCs may discriminate aGVHD from systemic CMV infections (Figure 4).

## 4. Discussion

In this study, we demonstrated that the ELISPOT assay might be useful for diagnosis and monitoring of aGVHD after HSCT. The assay reflects *in vivo* immune status since this assay does not require *in vitro* expansion or activation of specific T cells before testing. Our results demonstrated that the numbers of IFN-γ and IL-4 SFCs in patients with grade II-IV aGVHD were significantly higher than those in patients with grade 0 and/or I aGVHD. These results suggested that not only type 1 cells but also type 2 cells are involved during aGVHD, regardless of whether the type 2 cells promote or suppress the disease. This study has also shown that enumeration of IFN-γ and IL-4 SFCs may discriminate aGVHD from CMV infections. Thus, the ELISPOT assay may be an efficacious method for evaluating aGVHD.

Acute GVHD is the major complication after HSCT [24]. It has been postulated that cytokines produced by type 1 cells would augment the T-cell responses against the host, thereby leading to greater aGVHD in the recipients [3]. Type 2 cytokines can inhibit the production of proinflammatory cytokines [24]. Clinical and preclinical data tend to support this phenomenon [25]. However, some preclinical models have demonstrated protective effects of type 1 cytokines in aGVHD, although the timing, dose, route of administration, and the murine strain combination may be critical in determining their effect [26]. Moreover, as with the type 1 cytokines, there have been conflicting reports demonstrating that type 2 cytokines can worsen the outcome of aGVHD [27]. Therefore, with regard to the type 1/type 2 paradigm, it is not yet determined whether a particular pathway is protective or deleterious for aGVHD. In the current study, both type 1 (IFN-γ) and type 2 (IL-4) SFCs were involved in aGVHD following HSCT (Figure 1).

IL-10, encoded by the *IL10* gene, is a known anti-inflammatory cytokine. Lin *et al*. described the association between the recipient’s IL-10 promoter polymorphism and the risk of aGVHD [9]. IL-10 could reduce immune responsiveness and suppress aGVHD [28]. In the current study, there was no significant correlation between the number of IL-10 SFCs and activity of aGVHD, although IL-10 SFCs in patients with aGVHD, regardless of the severity, increased at 3 and 6 months after HSCT. Therefore, these results might be reflective of the regulatory function of IL-10 because IL-10 production may not be in parallel with aGVHD.

Recently, it has been clearly shown that a newly identified subset of IL-17-producing CD4^+^ T-cells, named Th-17 cells [29], play a crucial role in orchestrating the immune reactions that trigger inflammation [30]. Dander *et al*. reported that an increased Th17 population of peripheral blood was observed in patients with aGVHD [31]. In the current study, the number of IL-17 SFCs was not increased in patients with or without aGVHD although PMA-activated PBMC controls were used for verifying the assay. These data do not contradict the previous data. Unlike our report using unstimulated cells for the ELISPOT assay, Dander’s report used the cells stimulated with PMA and ionomycin prior to the ELISPOT assay and unstimulated cells were used as negative controls.

There is a possibility that the numbers of cytokine SFCs are affected by immunosuppressive drugs, including cyclosporine A, tacrolimus, methotrexate, and corticosteroid for prophylaxis and/or treatment of aGVHD. In fact, most of our patients (86%) with grade I-IV aGVHD were placed on one or more of these immunosuppressants when cytokine SFCs were examined. However, these immunosuppressants do not affect the balance between type 1 and type 2 cells [32,33]. Therefore, the current *in vitro* data reflected the *in vivo* immune status without skewing the type 1/type 2 balance by immunosuppressants used.

Infection also affects type 1 and/or type 2 cytokine productions. CMV infection is able to trigger IFN-γ and IL-4 response [34] and elicit potent memory responses in chronically infected immunocompetent hosts [35]. However, impaired CMV-specific immunity may persist in the first 100 days after HSCT [36,37]. In the current study, we could not detect the elevation of IFN-γ and IL-4 SFCs in patients with CMV infection without aGVHD (Figure 4). Thus, the ELISPOT assay can discriminate aGVHD from systemic CMV infection.

As for the surface phenotypes (type 1, type 2, regulatory T-cell type, and Th-17 cell type) of SFCs in the current study, we did not determine cell types partly because the available cell number was limited. Nevertheless, SFCs are likely to contain predominantly CD8^+^ T cells at the times when SFCs were examined, since the normalization of CD8^+^ T cells is more rapid than that of CD4^+^ T cells. Total T cells and CD4^+^ T cells are characteristically low in numbers until between 6 and 12 months post-transplant, while CD8^+^ T cells return quickly to normal range and often remain elevated long term post-transplant [38]. It has been reported that CD8^+^ T cells produce IFN-γ, IL-4, and IL-10 [39,40]. Therefore, it is likely that SFCs containing CD8^+^ T cells produced these cytokines, although CD4^+^ T cells (especially regulatory T cells), natural killer cells, mast cells, monocytes, and macrophages may also participate in cytokine production.

## 5. Conclusions

The enumeration of cytokine SFCs is readily available and a valuable clinical aid in the evaluation of HSCT patients, especially when complicated with systemic infections in the posttransplant period. IFN-γ and IL-4 SFCs may help to discriminate aGVHD from systemic infections. Larger studies are needed to confirm the current findings.

## References

[1] Mitchell A.E., Derrington P., Turner P., Hunt L.P., Oakhill A., Marks D.I. (2004). Gram-negative bacteraemia (GNB) after 428 unrelated donor bone marrow transplants (UD-BMT): Risk factors, prophylaxis, therapy and outcome. Bone Marrow Transplant..

[2] Cooke K.R., Gerbitz A., Crawford J.M., Teshima T., Hill G.R., Tesolin A., Rossignol D.P., Ferrara J.L. (2001). LPS antagonism reduces graft-*versus*-host disease and preserves graft-*versus*-leukemia activity after experimental bone marrow transplantation. J. Clin. Invest..

[3] Krenger W., Ferrara J.L. (1996). Graft-*versus*-host disease and the Th1/Th2 paradigm. Immunol. Res..

[4] Liem L.M., van Lopik T., van Nieuwenhuijze A.E., van Houwelingen H.C., Aarden L., Goulmy E. (1998). Soluble Fas levels in sera of bone marrow transplantation recipients are increased during acute graft-*versus*-host disease but not during infections. Blood.

[5] Fujimori Y., Takatsuka H., Takemoto Y., Hara H., Okamura H., Nakanishi K., Kakishita E. (2000). Elevated interleukin (IL)-18 levels during acute graft-*versus*-host disease after allogeneic bone marrow transplantation. Br. J. Haematol..

[6] Visentainer J.E., Lieber S.R., Persoli L.B., Vigorito A.C., Aranha F.J., de Brito Eid K.A., Oliveira G.B., Miranda E.C., de Souza C.A. (2003). Serum cytokine levels and acute graft-*versus*-host disease after HLA-identical hematopoietic stem cell transplantation. Exp. Hematol..

[7] Hori T., Naishiro Y., Sohma H., Suzuki N., Hatakeyama N., Yamamoto M., Sonoda T., Mizue Y., Imai K., Tsutsumi H., Kokai Y. (2008). CCL8 is a potential molecular candidate for the diagnosis of graft-*versus*-host disease. Blood.

[8] Paczesny S., Krijanovski O.I., Braun T.M., Choi S.W., Clouthier S.G., Kuick R., Misek D.E., Cooke K.R., Kitko C.L., Weyand A., Bickley D., Jones D., Whitfield J., Reddy P., Levine J.E., Hanash S.M., Ferrara J.L. (2009). A biomarker panel for acute graft-*versus*-host disease. Blood.

[9] Lin M.T., Storer B., Martin P.J., Tseng L.H., Gooley T., Chen P.J., Hansen J.A. (2003). Relation of an interleukin-10 promoter polymorphism to graft-*versus*-host disease and survival after hematopoietic-cell transplantation. New Engl. J. Med..

[10] Hattori H., Matsuzaki A., Suminoe A., Ihara K., Nagatoshi Y., Sakata N., Kawa K., Okamura J., Hara T. (2002). Polymorphisms of transforming growth factor-beta1 and transforming growth factor-beta1 type II receptor genes are associated with acute graft-*versus*-host disease in children with HLA-matched sibling bone marrow transplantation. Bone Marrow Transplant..

[11] Theobald M., Nierle T., Bunjes D., Arnold R., Heimpel H. (1992). Host-specific interleukin-2-secreting donor T-cell precursors as predictors of acute graft-*versus*-host disease in bone marrow transplantation between HLA-identical siblings. New Engl. J. Med..

[12] Hempel L., Korholz D., Nussbaum P., Bonig H., Burdach S., Zintl F. (1997). High interleukin-10 serum levels are associated with fatal outcome in patients after bone marrow transplantation. Bone Marrow Transplant..

[13] El Kassar N., Legouvello S., Joseph C.M., Salesses P., Rieux C., Cordonnier C., Vernant J.P., Farcet J.P., Bierling P., Kuentz M. (2001). High resolution HLA class I and II typing and CTLp frequency in unrelated donor transplantation: A single-institution retrospective study of 69 BMTs. Bone Marrow Transplant..

[14] Tanguay S., Killion J.J. (1994). Direct comparison of ELISPOT and ELISA-based assays for detection of individual cytokine-secreting cells. Lymphokine Cytokine Res..

[15] Han P., Hodge G. (1999). Intracellular cytokine production and cytokine receptor interaction of cord mononuclear cells: Relevance to cord blood transplantation. Br. J. Haematol..

[16] Glucksberg H., Storb R., Fefer A., Buckner C.D., Neiman P.E., Clift R.A., Lerner K.G., Thomas E.D. (1974). Clinical manifestations of graft-*versus*-host disease in human recipients of marrow from HL-A-matched sibling donors. Transplantation.

[17] Gondo H., Minematsu T., Harada M., Akashi K., Hayashi S., Taniguchi S., Yamasaki K., Shibuya T., Takamatsu Y., Teshima T. (1994). Cytomegalovirus (CMV) antigenaemia for rapid diagnosis and monitoring of CMV-associated disease after bone marrow transplantation. Br. J. Haematol..

[18] Tanaka Y., Kanda Y., Kami M., Mori S., Hamaki T., Kusumi E., Miyakoshi S., Nannya Y., Chiba S., Arai Y., Mitani K., Hirai H., Mutou Y. (2002). Monitoring cytomegalovirus infection by antigenemia assay and two distinct plasma real-time PCR methods after hematopoietic stem cell transplantation. Bone Marrow Transplant..

[19] Kobayashi M., Azuma E., Ido M., Hirayama M., Jiang Q., Iwamoto S., Kumamoto T., Yamamoto H., Sakurai M., Komada Y. (2001). A pivotal role of Rho GTPase in the regulation of morphology and function of dendritic cells. J. Immunol..

[20] Hirayama M., Azuma E., Kumamoto T., Iwamoto S., Yamada H., Nashida Y., Araki M., Kageyama S., Tamaki S., Kawakami K., Yamamoto H., Komada Y. (2005). Prediction of acute graft-*versus*-host disease and detection of distinct end-organ targets by enumeration of peripheral blood cytokine spot-forming cells. Transplantation.

[21] Emery V.C., Sabin C.A., Cope A.V., Gor D., Hassan-Walker A.F., Griffiths P.D. (2000). Application of viral-load kinetics to identify patients who develop cytomegalovirus disease after transplantation. Lancet.

[22] Li C.R., Greenberg P.D., Gilbert M.J., Goodrich J.M., Riddell S.R. (1994). Recovery of HLA-restricted cytomegalovirus (CMV)-specific T-cell responses after allogeneic bone marrow transplant: Correlation with CMV disease and effect of ganciclovir prophylaxis. Blood.

[23] Ljungman P., Aschan J., Lewensohn-Fuchs I., Carlens S., Larsson K., Lonnqvist B., Mattsson J., Sparrelid E., Winiarski J., Ringden O. (1998). Results of different strategies for reducing cytomegalovirus-associated mortality in allogeneic stem cell transplant recipients. Transplantation.

[24] Ferrara J.L., Cooke K.R., Pan L., Krenger W. (1996). The immunopathophysiology of acute graft-*versus*-host-disease. Stem Cells.

[25] Williamson E., Garside P., Bradley J.A., More I.A., Mowat A.M. (1997). Neutralizing IL-12 during induction of murine acute graft-*versus*-host disease polarizes the cytokine profile toward a Th2-type alloimmune response and confers long term protection from disease. J. Immunol..

[26] Blazar B.R., Taylor P.A., Panoskaltsis-Mortari A., Vallera D.A. (1998). Rapamycin inhibits the generation of graft-*versus*-host disease- and graft-*versus*-leukemia-causing T cells by interfering with the production of Th1 or Th1 cytotoxic cytokines. J. Immunol..

[27] Murphy W.J., Welniak L.A., Taub D.D., Wiltrout R.H., Taylor P.A., Vallera D.A., Kopf M., Young H., Longo D.L., Blazar B.R. (1998). Differential effects of the absence of interferon-gamma and IL-4 in acute graft-*versus*-host disease after allogeneic bone marrow transplantation in mice. J. Clin. Invest..

[28] Weston L.E., Geczy A.F., Briscoe H. (2006). Production of IL-10 by alloreactive sibling donor cells and its influence on the development of acute GVHD. Bone Marrow Transplant..

[29] Harrington L.E., Hatton R.D., Mangan P.R., Turner H., Murphy T.L., Murphy K.M., Weaver C.T. (2005). Interleukin 17-producing CD4+ effector T cells develop via a lineage distinct from the T helper type 1 and 2 lineages. Nat. Immunol..

[30] Annunziato F., Cosmi L., Santarlasci V., Maggi L., Liotta F., Mazzinghi B., Parente E., Fili L., Ferri S., Frosali F., Giudici F., Romagnani P., Parronchi P., Tonelli F., Maggi E., Romagnani S. (2007). Phenotypic and functional features of human Th17 cells. J. Exp. Med..

[31] Dander E., Balduzzi A., Zappa G., Lucchini G., Perseghin P., Andre V., Todisco E., Rahal D., Migliavacca M., Longoni D., Solinas G., Villa A., Berti E., Mina P.D., Parma M., Allavena P., Biagi E., Rovelli A., Biondi A., D’Amico G. (2009). Interleukin-17-producing T-helper cells as new potential player mediating graft-*versus*-host disease in patients undergoing allogeneic stem-cell transplantation. Transplantation.

[32] Sakuma S., Higashi Y., Sato N., Sasakawa T., Sengoku T., Ohkubo Y., Amaya T., Goto T. (2001). Tacrolimus suppressed the production of cytokines involved in atopic dermatitis by direct stimulation of human PBMC system. (Comparison with steroids). Int. Immunopharmacol..

[33] Rentenaar R.J., Heydendael V.M., Diepen F.N., Rie M.A., Berge I.J. (2004). Systemic treatment with either cyclosporin A or methotrexate does not influence the T Helper 1/t Helper 2 balance in psoriatic patients. J. Clin. Immunol..

[34] Wang Y.L., Zhang Y.Y., Zhou Y.L., Zhu Z.J., Tang Z.Q., Jiang Y., Peng L., Li G., Zhang X.H. (2004). T-helper and T-cytotoxic cell subsets monitoring during active cytomegalovirus infection in liver transplantation. Transplant. Proc..

[35] Bitmansour A.D., Waldrop S.L., Pitcher C.J., Khatamzas E., Kern F., Maino V.C., Picker L.J. (2001). Clonotypic structure of the human CD4+ memory T cell response to cytomegalovirus. J. Immunol..

[36] Cwynarski K., Ainsworth J., Cobbold M., Wagner S., Mahendra P., Apperley J., Goldman J., Craddock C., Moss P.A. (2001). Direct visualization of cytomegalovirus-specific T-cell reconstitution after allogeneic stem cell transplantation. Blood.

[37] Luo X.H., Huang X.J., Liu K.Y., Xu L.P., Liu D.H. (2010). Protective immunity transferred by infusion of cytomegalovirus-specific CD8(+) T cells within donor grafts: Its associations with cytomegalovirus reactivation following unmanipulated allogeneic hematopoietic stem cell transplantation. Biol. Blood Marrow Transplant..

[38] Atkinson K. (1990). Reconstruction of the haemopoietic and immune systems after marrow transplantation. Bone Marrow Transplant.

[39] Paganelli R., Scala E., Ansotegui I.J., Ausiello C.M., Halapi E., Fanales-Belasio E., D’Offizi G., Mezzaroma I., Pandolfi F., Fiorilli M., Cassone A., Aiuti F. (1995). CD8+ T lymphocytes provide helper activity for IgE synthesis in human immunodeficiency virus-infected patients with hyper-IgE. J. Exp. Med..

[40] Li L., Sad S., Kagi D., Mosmann T.R. (1997). CD8Tc1 and Tc2 cells secrete distinct cytokine patterns *in vitro* and in vivo but induce similar inflammatory reactions. J. Immunol..

